# Codon-optimized TDP-43 mediates neurodegeneration in a *Drosophila* model of ALS/FTLD

**DOI:** 10.3389/fgene.2023.881638

**Published:** 2023-03-09

**Authors:** Tanzeen Yusuff, Ya-Chu Chang, Tzu-Kang Sang, George R. Jackson, Shreyasi Chatterjee

**Affiliations:** ^1^ Department of Neuroscience and Cell Biology, University of Texas Medical Branch at Galveston, Galveston, TX, United States; ^2^ Mitchell Center for Neurodegenerative Diseases, University of Texas Medical Branch at Galveston, Galveston, TX, United States; ^3^ Department of Life Science, Institute of Biotechnology, National Tsing Hua University, Hsinchu, Taiwan; ^4^ Brain Research Center, National Tsing Hua University, Hsinchu, Taiwan; ^5^ Department of Neurology, University of Texas Medical Branch at Galveston, Galveston, TX, United States; ^6^ Department of Biochemistry and Molecular Biology, University of Texas Medical Branch at Galveston, Galveston, TX, United States; ^7^ Department of Neurology, Baylor College of Medicine, Houston, TX, United States; ^8^ National Parkinson’s Disease Research Education and Clinical Center, Michael E. DeBakey VA Medical Center, Houston, TX, United States; ^9^ Department of Biochemistry, School of Science and Technology, Nottingham Trent University, Nottingham, United Kingdom

**Keywords:** *Drosophila*, neurodegeneration, TDP-43, ALS, FTLD

## Abstract

Transactive response DNA binding protein-43 (TDP-43) is known to mediate neurodegeneration associated with amyotrophic lateral sclerosis (ALS) and frontotemporal lobar degeneration (FTLD). The exact mechanism by which TDP-43 exerts toxicity in the brains, spinal cord, and lower motor neurons of affected patients remains unclear. In a novel *Drosophila melanogaster* model, we report gain-of-function phenotypes due to misexpression of insect codon-optimized version of human wild-type TDP-43 (CO-TDP-43) using both the binary GAL4/*UAS* system and direct promoter fusion constructs. The CO-TDP-43 model showed robust tissue specific phenotypes in the adult eye, wing, and bristles in the notum. Compared to non-codon optimized transgenic flies, the CO-TDP-43 flies produced increased amount of high molecular weight protein, exhibited pathogenic phenotypes, and showed cytoplasmic aggregation with both nuclear and cytoplasmic expression of TDP-43. Further characterization of the adult retina showed a disruption in the morphology and function of the photoreceptor neurons with the presence of acidic vacuoles that are characteristic of autophagy. Based on our observations, we propose that TDP-43 has the propensity to form toxic protein aggregates *via* a gain-of-function mechanism, and such toxic overload leads to activation of protein degradation pathways such as autophagy. The novel codon optimized TDP-43 model is an excellent resource that could be used in genetic screens to identify and better understand the exact disease mechanism of TDP-43 proteinopathies and find potential therapeutic targets.

## Introduction

Transactive response DNA binding protein-43 (TDP-43), encoded by *TARDBP* gene in the human genome, has been identified as a major component for the pathology of motor neuron diseases and related neurodegenerative diseases (Neumann et al., 2006; Hasegawa et al., 2007). TDP-43 is a highly conserved and ubiquitously expressed protein that is primarily involved in regulation of RNA levels, RNA trafficking, and alternative splicing. The presence of tau-negative TDP-43 and ubiquitin-positive inclusion bodies is a major disease hallmark of Amyotrophic lateral sclerosis (ALS) and frontotemporal lobar degeneration (FTLD) (Arai et al., 2006; Neumann et al., 2006; Mackenzie and Rademakers, 2008; Lee et al., 2012). In the diseased state, TDP-43 is found to be ubiquitinated and phosphorylated and exhibits truncated C-terminal fragments and insoluble inclusions. The distinctive pathology of TDP-43 mediated neurodegeneration also involves its mislocalization to the cytoplasm and the loss of normal nuclear expression (Arai et al., 2009; Barmada et al., 2010; Guo et al., 2011; Lee et al., 2012; Nguyen et al., 2018). Mutations in the *TARDBP* gene are associated with both familial and sporadic cases of these diseases. Most of the dominant missense mutations are present in the glycine-rich domain near the C-terminal of TDP-43 (Nonaka et al., 2009; Lee et al., 2012), and have been linked to the formation of toxic TDP-43 aggregates that mediate neurodegeneration (Igaz et al., 2011). Protein-protein interactions, hyperphosphorylation, ubiquitination, and cleavage of the prion-like C-terminal fragment have been implicated in the formation of these TDP-43 aggregates (Johnson et al., 2009). In addition, the increased load of toxic protein aggregates has been suggested to cause defects in protein degradation systems, including autophagy and the ubiquitin proteasome system (UPS) (Rubinsztein, 2006; Blokhuis et al., 2013). In order to better understand the pathogenic mechanisms of TDP-43 mediated neurodegeneration, many cellular and animal models have been generated in both vertebrates and invertebrates, which include gain-of-function, RNA interference (RNAi) mediated suppression, and loss-of-function models (Johnson et al., 2008; Feiguin et al., 2009; Lu et al., 2009; Wegorzewska et al., 2009; Li et al., 2010; Stallings et al., 2010; Tsai et al., 2010; Estes et al., 2011; Gendron and Petrucelli, 2011; Romano et al., 2012; Vaccaro et al., 2012; Choksi et al., 2014).

*Drosophila melanogaster* has been widely utilized to study neurodegenerative diseases in an *in vivo* model system (Sang and Jackson, 2005; Chatterjee et al., 2009). We and others have previously shown that overexpressing toxic proteins such as full-length human tau, alpha-synuclein, or huntingtin in the *Drosophila* eye or neuromuscular junction results in degenerative phenotypes that are ideal for high-throughput screens, as well as for studying pathogenic mechanisms of the disease (Feany and Bender, 2000; Auluck et al., 2002; Shulman and Feany, 2003; Blard et al., 2007; Chatterjee et al., 2009; Wegorzewska et al., 2009; Li et al., 2010; Shulman et al., 2014). For example, loss-of-function models generated using deletion, non-sense or null mutations, and RNA-interference mediated knockdown of the *Drosophila* homolog of TDP-43, TBPH, showed shortened lifespan, locomotor and neuromuscular junction (NMJ) defects, and decreased dendritic branching of DA neurons (Feiguin et al., 2009; Lu et al., 2009). Furthermore, gain-of-function transgenic fly models overexpressing disease-specific variants of human TDP-43 (hTDP-43) showed decreased longevity, decreased locomotor activity, and increased morphological defects of motor neurons, along with axonal damage and, in some cases, neuronal loss (Lu et al., 2009; Hanson et al., 2010; Li et al., 2010; Ritson et al., 2010; Voigt et al., 2010; Estes et al., 2011; Guo et al., 2011; Li et al., 2011; Miguel et al., 2011; Langellotti et al., 2016; Chang and Morton, 2017; Pons et al., 2017). These gain-of-function mutations only account for about 10% of familial cases of ALS/FTLD, while 90% of affected individuals are sporadic cases involving wild-type TDP-43 mediated neurodegeneration (Nguyen et al., 2018). However, current studies involving wild-type TDP-43 have reported only subtle phenotypes that were difficult to quantify or did not exhibit robust disease-associated pathology. Therefore, there is a need for a robust model of wild-type TDP-43 mediated pathology to understand the cellular mechanisms associated with ALS/FTLD.

We generated an overexpression model of the human wild-type TDP-43 transgene by codon-optimization to accommodate insect transcriptional and translational machinery (Supplementary Figure S3). It has been shown, even in *D. melanogaster*, that certain 3-base pair sequences or codons in the mRNA transcript are more optimal in translating into the same amino acid over others (Powell and Moriyama, 1997; Welch et al., 2009). Using this phenomenon, we manipulated the human *TARDBP* gene by altering the coding region so that codons were optimized in a *Drosophila melanogaster* cellular environment to maximize TDP-43 expression, henceforth referred to as CO-TDP-43. In contrast to previous fly models, we demonstrate that the CO-TDP-43 lines lead to increased TDP-43 expression, hyperphosphorylation, and form toxic cytoplasmic aggregates that gives rise to strong phenotypes when expressed in the fly retina, wing, and notum. Further characterization of the retinal phenotype revealed a disruption in the internal morphology and function of the photoreceptor neurons, as well as presence of acidic autophagic-lysosomal vacuoles that are positive for key autophagy proteins. Our CO-TDP-43 model recapitulates phenotypes of ALS/FTLD disease pathology and is an ideal resource for investigating the mechanisms of pathogenesis for these diseases.

## Materials and methods

### Fly stocks and genetics

Codon optimized TDP-43 gene was synthesized from DNA2.0 (ATUM, Newark, CA, United States of America). The complete sequence of the codon optimized TDP-43 is provided in the Supplementary Figure S3. *Drosophila* kozak sequence (ATCAAC) was added upstream of the start codon for the TDP43 gene. These constructs were subcloned into the Not1-Xba1 site of the modified fly upstream activation sequence (*UAS*) expression (*pEx-UAS*) and *glass* (*pEx-gl*) vectors (Exelixis, San Francisco, CA, United States of America). The expression vectors containing the CO-TDP-43 gene were then microinjected into the flies to obtain transgenic flies (BestGene, Chino Hills, CA, United States of America). We generated multiple transgenic fly lines as noted in Supplementary Table S1. We selected *UAS-*TDP-43_6 line for our subsequent experiments with various GAL4 lines and the *gl-*CO-TDP-43_4 line for subsequent experiments using the eye as a model. The expression of non-CO-TDP-43 is driven in the fly eye by the *glass* multimer reporter, *GMR*-GAL4 on the X-chromosome (Freeman, 1996). All transgenic lines, both codon-optimized and non-codon optimized, express human wild-type TDP-43. Flies expressing human codon wild-type TDP-43 using the *UAS* promoter were obtained from Dr. Fen-Biao Gao (University of Massachusetts, Worcester, MA, United States of America) (Lu et al., 2009). *SevEP*-GAL4 driver (expressed in R7 and R8 photoreceptor neurons) was recombined with *UAS*-TDP-43CO to obtain stable transgenic flies expressing w^1118^;*SevEP*-GAL4,*UAS*-TDP-43CO/CyO; +. The *GMR*-GAL4 on the X-chromosome was placed in trans to the *gl*-TDP-43CO line to generate *GMR*-GAL4;*gl-*TDP-43CO/CyO transgenic flies. The following stocks were obtained from Bloomington *Drosophila* Stock Center (Bloomington, Indiana University, IN, United States of America): w^1118^;*UAS*-LacZ (BDSC 3955), w^1118^,*GMR*-myr-mRFP (BDSC 7121), y^1^,w^1118^;Sp/CyO; eGFP-ATG5 (recombined from BDSC 59848), y^1^,w^1118^;*UAS*-GFP-mCherry-ATG8 (BDSC 37749), *GMR*-GAL4(X) (eye specific) (BDSC 79572), w^1118^;*SevEP*-GAL4 (R7 and R8 in photoreceptor cells) (BDSC 5793), w^1118^,*beadex* ^MS1096^-GAL4 (wing driver) (BDSC 8860), w^1118^;*Scabrous*-GAL4 (sensory organ precursor and wing discs driver) (BDSC 6479), and y^1^,w^*^; *CCAP-*GAL4 (driver expressed in *CCAP*/bursicon neurons in ventral nerve cord and subesophageal ganglion in adult brain) (BDSC 25685). *Eq*-GAL4 (bristle driver) and y^1^,w^1118^;*Rh1*-GAL4/CyO (expressed in R1-R6 photoreceptor cells) were obtained from Dr. Hugo J. Bellen (Baylor College of Medicine, Houston, TX). All crosses were set and flies were maintained at room temperature (22°C) in standard *D. melanogaster* Jazzmix medium (Applied Scientific, Fisher Scientific, Pittsburgh, PA, United States of America).

### Immunohistochemistry

Adult retina and imaginal eye discs from third instar larvae were dissected and fixed in 4% paraformaldehyde for 1 h on ice. Adult retina was washed in 0.5% PTX for 3 h to reduce autofluorescence. The tissues were blocked in 0.8% PBS + Triton-X + BSA for 2 h and incubated with primary antibody overnight at 4°C. The tissues were incubated in secondary antibody for 2 h at room temperature, washed in 0.1% PBS + Triton-X and mounted on glass slides with Vectashield (Vector Laboratories, Burlingame, CA, United States). Tissues were stained with the following antibodies: mouse monoclonal anti-TDP-43 antibody (1:500, Abcam, Cambridge, MA, United States), rabbit polyclonal anti-TDP-43 antibody (1:500, Proteintech, Chicago, IL,United States), rat monoclonal anti-Elav (1:20, DSHB, University of Iowa, Iowa City, IA, United States), mouse monoclonal anti-GFP (1:400, Millipore, Billerica, MA, United States), Alexa Fluor 633-conjugated Phalloidin (1:30, Invitrogen, Grand Island, NY, United States), Alexa Fluor 488 conjugated chicken anti-rat (1:400, Invitrogen, Grand Island, NY, United States) Alexa Fluor 568 conjugated goat anti-rabbit (1:400, Invitrogen, Grand Island, NY, United States) and Alexa Fluor 568 conjugated goat anti-mouse (1:400, Invitrogen, Grand Island, NY, United States).

### Immunoblotting

Overexpression of CO-TDP-43 in the fly eye was used to measure total protein levels by immunoblotting. Approximately 50 fly heads were decapitated and homogenized for 1 min in homogenization buffer (10 mM Tris-HCl, 0.8 M NaCl, 1 mM EGTA, pH 8.0% and 10% sucrose) along with 1X PhosSTOP phosphatase and 1X cOmplete protease buffer (Roche Applied Science, Indianapolis, IN, United States). The homogenized samples were centrifuged at 4 °C for 15 min at 18,000 g. The supernatant was collected and equal parts of the supernatant and Laemmle sample loading buffer with β-mercaptoethanol (Bio-Rad, Hercules, CA, United States) was added for each sample. Following a brief pulse centrifugation, samples were loaded on 4%–20% SDS-PAGE gels (Bio-Rad, Hercules, CA, United States) for electrophoresis. For higher molecular weight species detection, the fly heads were homogenized in 1X PBS along with the same protease and phosphatase inhibitors. Non-reducing sample loading buffer (Nupage sample buffer, Life Sciences, Grand Island, NY, United States) was added to the supernatant without β-mercaptoethanol. The blots were blocked in 5% milk, incubated with primary antibodies overnight at 4 °C, washed in 1X TBS + Tween, and incubated with secondary antibody for 1 h at room temperature. The following antibodies were used: Mouse monoclonal anti-TDP-43 antibody (1:1,000, Abcam), Rabbit Polyclonal anti-TDP43 antibody (1:1,000, Proteintech), Rabbit monoclonal anti-Phospho-TDP43 (1:500, Ser 409/410, Proteintech), Mouse monoclonal anti-tubulin antibody (1:1,000, DSHB, University of Iowa), Mouse monoclonal anti-beta Actin antibody (1:5,000, Santa Cruz Biotechnology), secondary anti-mouse IgG-HRP (1:2000, GE Healthcare), and anti-rabbit IgG-HRP (1:5,000, Sigma).

### Lysotracker staining

For Lysotracker staining, imaginal eye discs from the third instar larvae were dissected in 1X PBS solution without fixative. The eye discs were then stained with 100 nM LysoTracker Red DND-99 (Invitrogen) for 2 min, followed by a 1-min wash in 1X PBS. The tissues were mounted on a glass slide with a drop of 1X PBS solution; no Vectashield was added. The coverslip was sealed with nail polish and visualized immediately using a confocal microscope. The z-stack images were analyzed using the ImageJ software (Schneider et al., 2012).

### Electroretinogram

ERG was recorded in 1-day old flies using the same methods as previously described (Fabian-Fine et al., 2003; Williamson et al., 2010). Briefly, flies were glued on glass slides using Elmer’s non-toxic glue. Both the reference and recording electrodes were made of glass pipettes filled with 3 M KCl. The light stimulus was computer-controlled using white light-emitting diode system (MC1500; Schott), and was provided in 1-s pulses. The data was recorded using Clampex software (version 10.1; Axon Instruments) and measured and analyzed using Clampfit software (version 10.2; Axon Instruments).

### Microscopy

The adult eye, wing and bristle pictures were taken with a Nikon AZ100 M microscope equipped with a Nikon DS-Fi1 digital camera (Nikon Instruments, Melville, NY, United States). Extended depth of focus (EDF) and volumetric images were taken using the Nikon NIS-Elements AR 3.0 software as previously described (Ambegaokar and Jackson, 2011). The scanning electron microscopy (SEM) images were taken using JSM-6510LV SEM (JEOL United States, Peabody, MA, United States). The confocal images were taken with a Zeiss LSM 510 UV META laser scanning confocal microscope using 40X water and 63X oil-immersion high-resolution objectives. These images were analyzed using the LSM Image Browser and NIH ImageJ software (Schneider et al., 2012).

### Statistical analysis

The SEM and light-microscopy images were quantitated by image-J. Briefly, the area of the eye surfaces were measured by NIH image-J software and paired *t*-test was performed between the Control vs non-CO-TDP-43 vs CO-TDP-43 eyes for statistical analysis. Western Blot data comparing non-CO-TDP-43 and CO-TDP-43 was estimated by paired *t*-test. The histograms represent mean ± SEM and are analyzed using Graphpad prism software. Quantification of Lysotracker staining was performed using the NIH Image J software (Schneider et al., 2012). The measurements and histograms represent mean ± SEM and plotted using Microsoft Excel and SigmaPlot (version 10.1) software. Statistical analysis was performed using one-way ANOVA with Bonferroni’s correction and paired Student’s t-test with two-tailed distributions of equal variance.

### Data availability

The codon-optimized TDP-43 fly lines are available upon request.

## Results

### Codon-optimized wild-type TDP-43 flies exhibit an age-dependent robust eye phenotype

**FIGURE 1:**
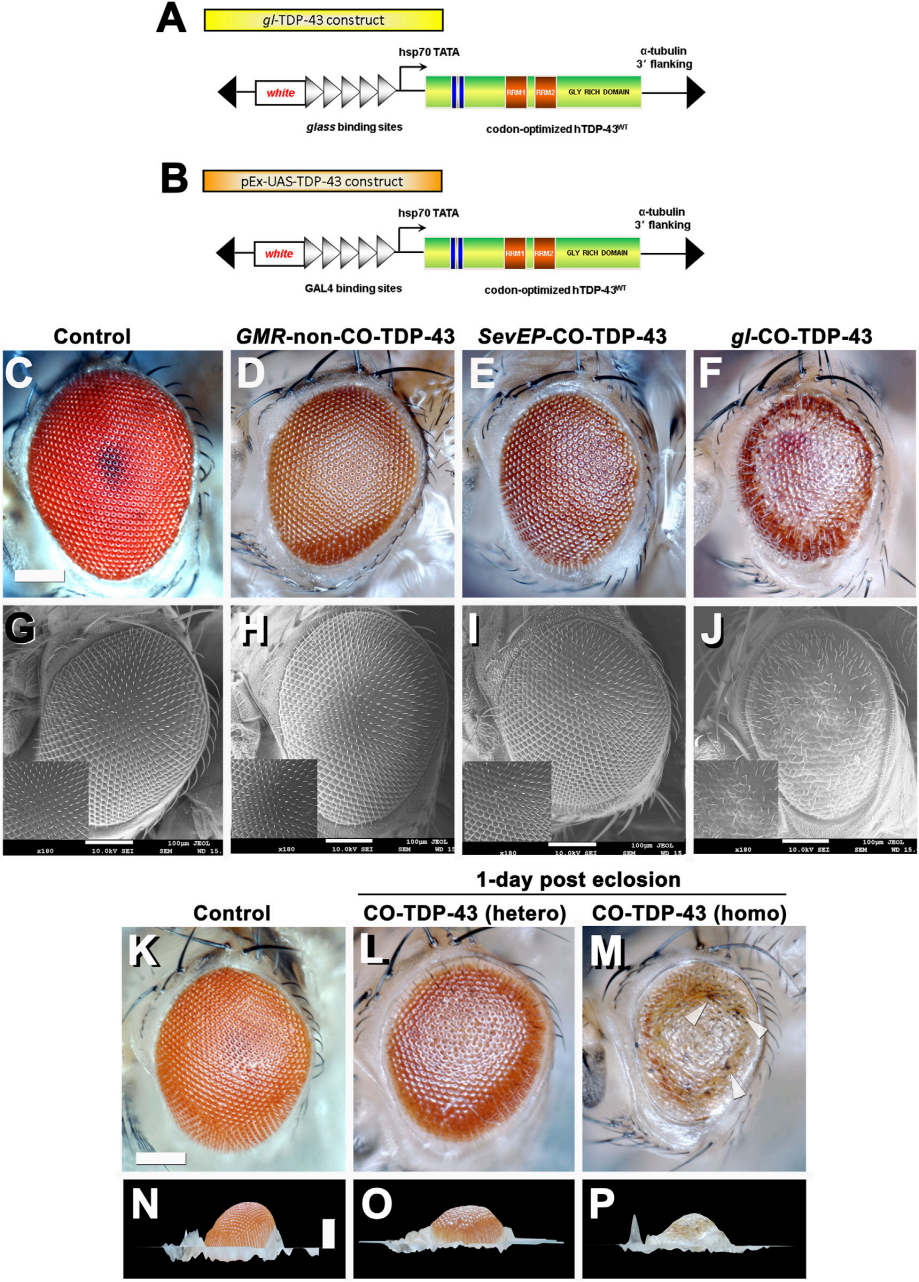
Misexpression of codon optimized TDP-43 induces depigmentation and irregularities in bristles compared to existing human wild-type TDP-43 lines. Transgenic flies stably express wild-type or codon optimized TDP-43 using eye promoters. All stocks were kept and maintained at room temperature (22°C). **(A, B)** Schematic of the CO-TDP-43 constructs using *glass* direct fusion promoter vector and UAS vector used to create the transgenic CO-TDP-43 lines. **(C–J)** Photomicrograph and scanning electron microscopy (SEM) images of the adult retina at 10-days post-eclosion (scale bar 100 uM). Compared to existing human wild-type non-CO-TDP-43 transgenic flies expressed using *GMR*-GAL4 promoter **(D, H)**, the CO-TDP-43 flies using *gl*ass direct fusion promoter **(F, J)** exhibit a robust eye phenotype including depigmentation, disruption in planar polarity and loss of bristles. *SevEP*-GAL4, a selective R7 and R8 photoreceptor neuron driver, recombined with CO-TDP-43 **(E, I)** also shows the same phenotypes. **(C, G)** are controls. **(K–P)** The robust phenotype mediated by CO-TDP-43 is both age and dosage dependent. At 1-day post-eclosion, CO-TDP-43 **(L)** shows less depigmentation compared to 10-days post-eclosion **(F)**. A homozygous CO-TDP-43 expression **(M)** shows a dramatically more robust phenotype with some necrosis (white arrowheads) at 1-day post-eclosion. Compared to control flies **(K, N)**, both hetero- and homozygous CO-TDP-43 shows decreased volume **(O, P)**. Scale bar: 100 nm. Genotypes: **(C)** Canton S, **(G)** w^1118^;*GMR*-GAL4/+, **(D, H)** w^1118^/+;*GMR*-GAL4/+;*UAS*-hTDP-43^WT^/+, **(E, I)** w^1118^/+;*SevEP*-GAL4,*UAS*-TDP-43^CO^/+; +, **(F, J)** w^1118^/+;*gl*-TDP-43^CO^/+; +, **(K, N)** Canton S, **(L, O)** w^1118^/+;*gl*-TDP-43^CO^/+; +, **(M, P)** w^1118^;*gl*-TDP-43^CO^;+.

We generated multiple codon-optimized CO-TDP-43 transgenic fly lines to investigate TDP-43 mediated neurodegeneration. We utilized both the yeast GAL4/*UAS* binary system (Brand and Perrimon, 1993) and a *glass (gl)* promoter direct fusion construct specifically generated to study TDP-43 mediated effects on the fly retina (Figures 1A, B). In addition, we also used another eye promoter, *Sevenless* (*SevEP*-GAL4), that only expresses in a subset of photoreceptor neurons (R7 and R8) and cone cells (Therrien et al., 1999). To highlight the robust effect observed in our CO-TDP-43 lines, we compared the phenotypes to a previously reported human TDP-43 transgenic line, which we denote as non-CO-TDP-43 (Lu et al., 2009; Choksi et al., 2014).

Heterozygous expression of CO-TDP-43 using the *gl* promoter caused depigmentation, roughness, disruption of polarity, and loss of inter-ommatidial bristles (Figures 1F, J). The CO-TDP-43 expressed using *SevEP*-GAL4 showed a similar but milder phenotype of the eye (Figures 1E, I). In comparison to the CO-TDP-43 flies, the non-CO-TDP-43 transgenic flies (Figures 1D, H) did not show a robust eye phenotype and appeared to be similar in morphology to the wild-type control flies (Figures 1C, G). Interestingly, the eye phenotype observed with heterozygous *gl*-CO-TDP-43 flies were age dependent. At 1-day post-eclosion, CO-TDP-43 exhibited a mild phenotype (Figure 1L) that worsened by 10-days (Figure 1F and Supplementary Figure S4). In contrast, flies with two copies of the CO-TDP-43 transgene showed a strong phenotype at 1-day post-eclosion, with apparent necrotic patches or hyperpigmentation (Figure 1M white arrowheads, compared to control in Figure 1K). In addition, the CO-TDP-43 flies with either one or two copies of the TDP-43 transgene showed less eye volume than wild-type control flies at 1-day post-eclosion (Figures 1O, P compared to control in Figure 1N). We were unable to observe these phenotypic differences in flies aged longer than 1-day post-eclosion as flies with two copies of the CO-TDP-43 transgene do not survive a day or two after eclosion. We also found that overexpression of CO-TDP-43 using *GMR*-GAL4 driver led to pupal lethality at 18°C and 25°C (Supplementary Table S1), with some escapers at 18 °C that showed necrotic patches (Supplementary Figure S1). Taken together, our results showed that CO-TDP-43 transgenic flies have a more robust eye phenotype indicative of neurodegeneration in retinal cells compared to non-CO-TDP-43 transgenic flies.

### Misexpression of codon-optimized wild-type TDP-43 leads to necrosis and severe phenotypes in wings and notum

**FIGURE 2:**
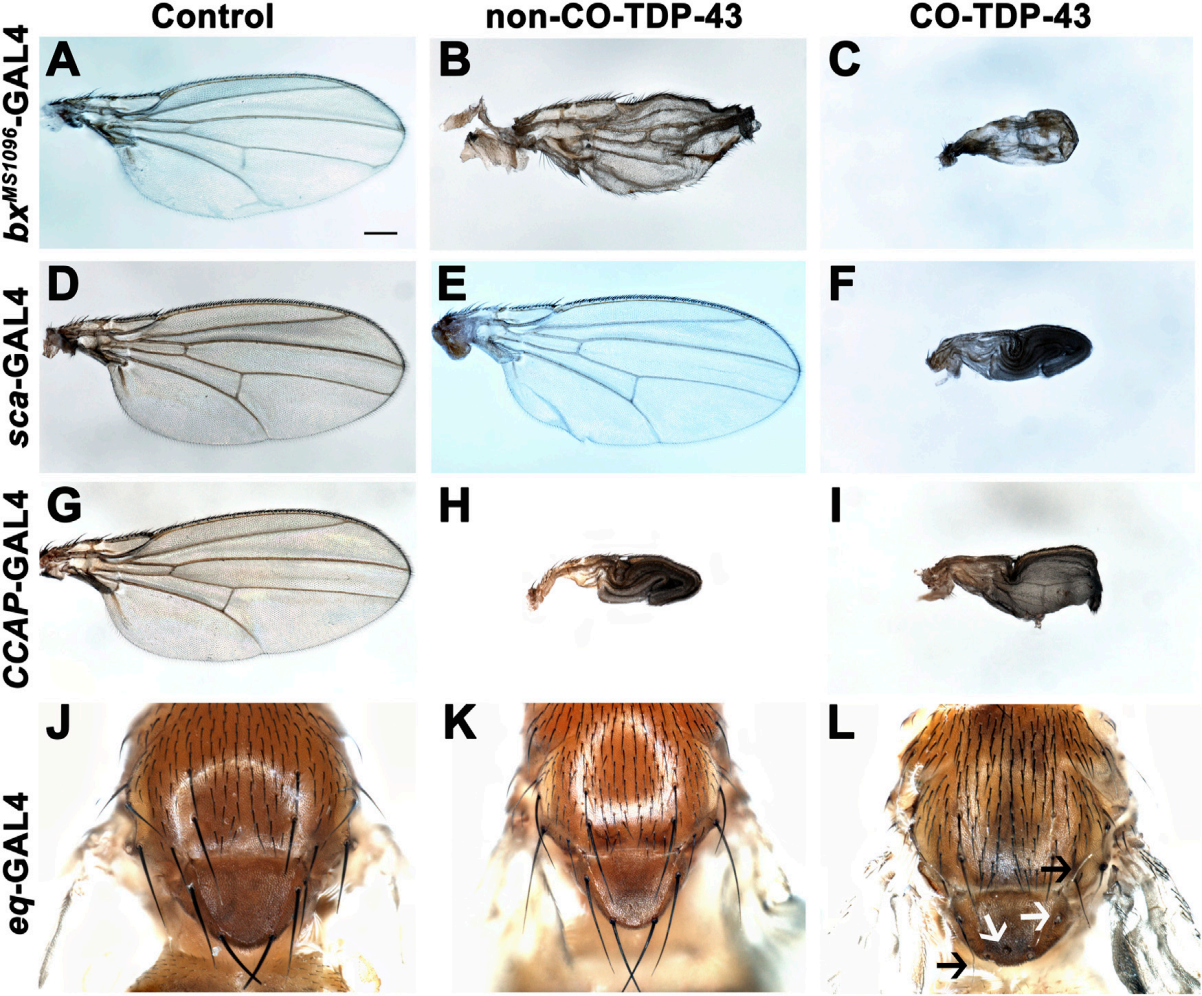
Misexpression of codon optimized TDP-43 leads to wing expansion and swelling defects as well as singed and loss of bristles in the fly notum. **(A–C)** CO-TDP-43 expressed in the wings using the wing-specific driver *bx* ^*MS1096*^-GAL4 leads to pharate adults with smaller, swollen and necrotic wings **(C)**, as compared to healthy, viable adults expressing human wild-type non-CO-TDP-43 with crumpled wings **(B)** and normal wings with the driver alone **(A)**. **(D–F)** Using *sca*-GAL4 driver, human wild-type non-CO-TDP-43 flies **(E)** have normal wings, similar to controls with the driver alone **(D)**, while CO-TDP-43 causes pharate adults with smaller, necrotic wings with expansion defect **(F)**. **(G–I)** *CCAP*-GAL4, expressed in CCAP/bursicon neurons in the ventral nerve cord and the subesophageal ganglion in the adult brain, driven expression of CO-TDP-43 **(I)** as well as human wild-type non-CO-TDP-43 **(H)** also exhibit similar wing expansion defects, compared to the driver alone **(G)**. **(J–L)** A bristle specific driver, *eq*-GAL4, causes a dramatic loss of bristles (white arrows) and singed bristles (black arrows) with CO-TDP-43 flies **(L)**, while human wild-type non-CO-TDP-43 **(K)** and driver alone **(J)** develop normal bristles. Scale bar: 200um. Genotypes: **(A)** w^1118^,*bx* ^*MS1096*^-GAL4/+; +;+, **(B)** w^1118^,*bx* ^*MS1096*^-GAL4/+; +;UAS-hTDP-43^WT^/+, **(C)** w^1118^,*bx* ^*MS1096*^-GAL4/+; +;UAS-TDP-43^CO^/+, **(D)** w^1118^/+;*sca*-GAL4/+; +, **(E)** w^1118^/+;*sca*-GAL4/+; UAS-hTDP-43^WT^/+, **(F)** w^1118^/+;*sca*-GAL4/+; UAS-TDP-43^CO^/+, **(G)** y^1^,w^*^/+;*CCAP*-GAL4/+; +, **(H)** y^1^,w^*^/+;*CCAP*-GAL4/+; UAS-hTDP-43^WT^/+, **(I)** y^1^,w^*^/+;*CCAP*-GAL4/+; UAS-TDP-43^CO^/+, **(J)** w^1118^/+; *eq*-GAL4/+; +;+, **(K)** w^1118^/+; *eq*-GAL4/+; +;UAS-hTDP-43^WT^/+, **(L)** w^1118^/+; *eq*-GAL4/+; +; UAS-TDP-43^CO^/+.

TDP-43 associated pathology in ALS patients have been linked to significant neuronal loss and early axonal atrophy in sensory nerves (Heads et al., 1991; Mochizuki et al., 2011). For example, Vaughan and colleagues reported that the pathogenic A315T mutation in TDP-43 affects neurite growth and decreased dendritic branching of sensory neurons (Vaughan et al., 2018). In fact, previously reported *Drosophila* neurodegeneration models showed that overexpression of the neurotoxic ataxin-1 mutant in sensory precursors using the *scabrous*-GAL4 (*sca*-GAL4) driver leads to loss of bristles in the adult fly (Tsuda et al., 2005). Similarly, we previously showed that misexpression of fly dVAP33, a gene linked to ALS, using *sca*-GAL4 leads to loss of notal macrochaetae (Ratnaparkhi et al., 2008). To further investigate the phenotypic effects of CO-TDP-43 on sensory precursor cells of the wing and notum, we used multiple wing and bristle drivers to misexpress TDP-43 protein, including *beadex* ^*MS1096*^-GAL4 (*bx* ^*MS1096*^-GAL4), *sca*-GAL4, *equate*-GAL4 (*eq*-GAL4), and *CCAP*-GAL4. We found that non-CO-TDP-43 transgene expressed using *bx* ^*MS1096*^-GAL4 led to viable adults with shriveled wings, with some flies having wings that were either necrotic or had areas of hyperpigmentation (Figure 2B). In contrast, CO-TDP-43 flies using the same driver exhibited a more severe phenotype, with pharate adults and very small and severely malformed wings with necrotic or hypermelanized patches (Figure 2C, compared to control in Figure 2A). Interestingly, unlike the previously reported model of ALS, neither non-CO-TDP-43 nor CO-TDP-43 had any effect on macrochaetae (bristles) on the notum when misexpressed using *sca*-GAL4 driver. Instead, the CO-TDP-43 transgenic flies produced pharate adults with necrotic wings that were unable to expand (Figure 2F, compared to non-CO-TDP-43 in Figure 2E and control in Figure 2D). Since we failed to see an effect of TDP-43 on macrochaetae using *sca*-GAL4, we used another bristle-specific driver, *eq*-GAL4, to misexpress non-CO and CO-TDP-43 in the fly notum (Tang and Sun, 2002). While both control and non-CO-TDP-43 flies showed normal macrochaetae formation (Figures 2J, K), CO-TDP-43 showed a dramatic loss of defective notal macrochaetae (Figure 2L). Furthermore, Vanden Broeck and colleagues previously showed that both up and downregulation of fly dTDP-43 cause selective apoptosis in the crustacean cardioactive peptide (CCAP)/bursicon neurons (Vanden Broeck et al., 2013). Loss of CCAP/bursicon neurons have been shown to cause pupal lethality with escapers that show wing expansion defect phenotypes (Park et al., 2003). Upon expression of non-CO-TDP-43 in the CCAP/bursicon neurons using *CCAP*-GAL4, we observed a similar wing expansion defect in adults (Figure 2H). Misexpression of CO-TDP-43 in CCAP/bursicon neurons resulted in smaller, necrotic and swollen wings compared to control flies (Figure 2I and Figure 2G, respectively). In summary, these results suggest that misexpression of CO-TDP-43 in flies leads to smaller wings with abnormal morphology and macrochaetae irregularities compared to misexpression of non-CO-TDP-43.

### Increased expression of codon-optimized TDP-43 exhibits disease-specific cytoplasmic mislocalization and aggregation

**FIGURE 3:**
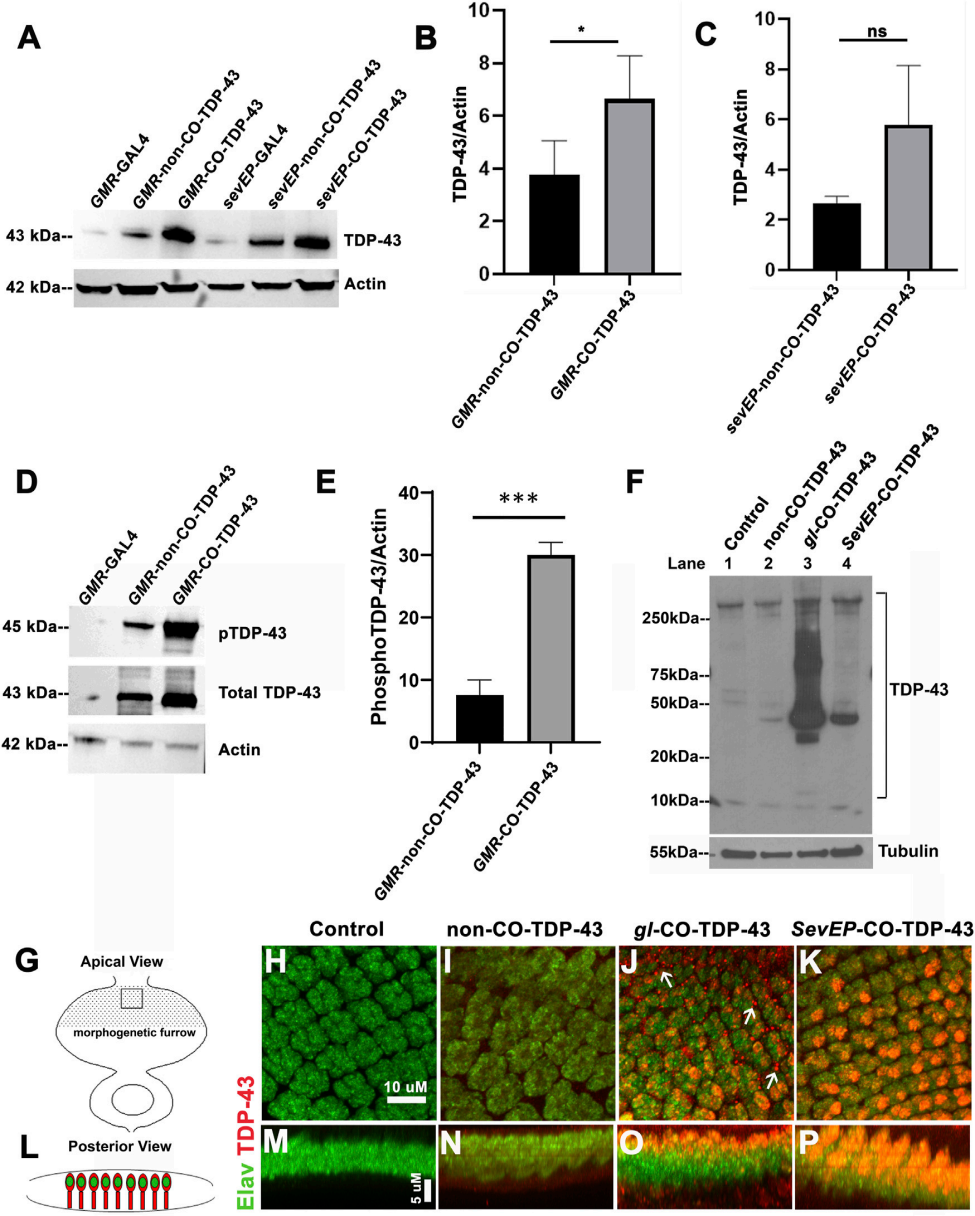
Codon optimized TDP-43 transgenic fly expresses higher total and phosphor-TDP43 protein levels, with robust mislocalization to the cytoplasm and aggregate formation in larval eye discs. **(A)** Western blot comparing the levels of total TDP-43 protein in the Controls vs CO-TDP-43 and non-CO-TDP-43 transgenics driven by the *GMR*-GAL4 and *SevEP*-GAL4 driver. These blots are probed with total TDP-43 antibody with Actin as the loading control. **(B, C)** Bar graphs displaying relative levels of TDP-43 derived by densitometric analysis from three separate blots. The level of expression is 2-fold higher in CO-TDP-43 transgenics compared to non-CO-TDP-43 lines in control of the *GMR*-GAL4 driver. Paired *t*-test between the transgenics shows a statistical significance *p < 0.02. There is also an increase in the case of CO-TDP-43 transgenics with *SevEP*-GAL4 compared to non-CO-TDP-43 flies, but this is not significant. **(D)** An enhanced level of phosphorylation at the Ser409/410 epitopes is found in the *GMR*-GAL4 driven CO-TDP-43 transgenics compared to non-CO-TDP-43 lines normalized to Actin levels. **(E)** Bar graphs displaying relative levels of phospho-TDP-43 phosphorylation derived by densitometric analysis. The level of expression is 3-fold higher in CO-TDP-43 transgenics compared to non-CO-TDP-43 lines using GMR-GAL4 driver. Paired *t*-test between the transgenics shows a statistical significance ***p < 0.0005. **(F)** CO-TDP-43 (*gl*-CO-TDP-43, lane 3) exhibits higher molecular weight species of TDP-43 as well as the known 35kD truncated c-terminal fragment compared to human wild-type non-CO-TDP-43 (lane 2) or the *SevEP*-GAL4 driven CO-TDP-43 (lane 4). Lane 1 is wild-type control. β-tubulin is presented as a loading control. **(G, L)** Schematic of the third instar larval imaginal eye-antennal disc from an apical and posterior view, respectively. The area represented with the rectangular box in **(G)** is the area imaged. **(H–P)** Confocal images of the third instar imaginal eye discs stained with neuronal marker *Elav* (green) and TDP-43 (red). There is a greater expression of both nuclear and cytoplasmic TDP-43 in CO-TDP-43 lines **(J, K)** as compared to human wild-type non-CO-TDP-43 **(I)**. The *gl*-CO-TDP-43 flies exhibit a more robust mislocalization and aggregation of cytoplasmic TDP-43; white arrows in **(J)**. **(H)** shows the control (scale bar 10 µm). **(M–P)** represents the posterior view of the eye discs to show nuclear and cytoplasmic TDP-43 expression (scale bar 5 µm). Genotypes: **(A)** w^1118^;*GMR*-GAL4/+, w^1118^;*GMR*-GAL4/+; UAS-hTDP-43^WT^/+, w^1118^;*GMR*-GAL4/+; UAS-hTDP-43^CO^, w^1118^; *SevEP*-GAL4/+, w^1118^;*SevEP*-GAL4/+; UAS-hTDP-43^WT^, w^1118^;*SevEP*-GAL4/+; UAS-hTDP-43^CO^ (in respective order) **(D)** w^1118^;*GMR*-GAL4/+, w^1118^;*GMR*-GAL4/+; UAS-hTDP-43^WT^/+, w^1118^;*GMR*-GAL4/+; UAS-hTDP-43^CO^ **(F)** w^1118^;+;+, w^1118^;*GMR*-GAL4/+; UAS-hTDP-43^WT^/+, w^1118^;*gl*-TDP-43^CO^/+; +, w^1118^;*SevEP*-GAL4,UAS-TDP-43^CO^/+; + (lane 1-4, respectively). **(H, M)** Canton S, **(I, N)** w^1118^; *GMR*-GAL4/+; UAS-hTDP-43^WT^/+, **(J, O)** w^1118^;*gl*-TDP-43^CO^/+; +, **(K, P)** w^1118^;*SevEP*-GAL4,UAS-TDP-43^CO^/+; +.

The robustness of the external phenotypes observed with CO-TDP-43 prompted us to examine the protein expression levels of the TDP-43 transgene in these flies. We next used multiple *gl* direct fusion CO-TDP-43 lines to examine TDP-43 expression levels in the fly eye. Compared to the *GMR*-GAL4 driven non-CO-TDP-43 transgenic flies, the CO-TDP-43 flies showed a 2-fold increase in monomeric total TDP-43 protein in multiple lines (Figures 3A, B and Supplementary Figure S2B, lane 5, 6 and 7). Next, we compared the TDP-43 protein levels between the CO-TDP-43 and non-CO-TDP-43 lines using the *SevEP* driver. In this case, the CO-TDP-43 protein levels were higher than the non-CO-TDP-43 lines, but this enhancement was not statistically significant (Figures 3A, C). Possibly because TDP-43 is expressed only in a subset of photoreceptor neurons. The line showing the highest increase in protein levels, one of the *gl* direct fusion CO-TDP-43 lines (Supplementary Figure S2B, lane 5), also demonstrated a robust eye phenotype (Figure 1) and was therefore used in subsequent experiments. Predictably, compared to the *gl*-CO-TDP43 line, the recombinant line using *SevEP*-GAL4 to overexpress CO-TDP-43 did not show an increase in total TDP-43 expression, since it is only expressed in a subset of photoreceptor neurons (Supplementary Figure S2B, lane 8). Next, we assessed the levels of TDP-43 phosphorylated at Serine 409/410 residues since phosphorylation of these residues are associated with TDP-43 oligomerization and toxicity (Gao et al., 2018). We found high levels of phospho-TDP43 in our CO-TDP-43 lines compared to non-CO-TDP-43 (Figures 3D, E).

As observed in patients with ALS and FTLD, high-molecular weight toxic species of TDP-43 have been detected in transgenic flies overexpressing TDP-43 containing pathogenic variants (Miguel et al., 2011; Chang and Morton, 2017). For example, we previously reported high-molecular weight species of TDP-43 in flies overexpressing disease-associated TDP-43 Q331K mutations (Choksi et al., 2014). To investigate if we are able to detect these high-molecular weight oligomeric species in our codon optimized lines, we used the *gl* direct fusion line and the *SevEP*-GAL4 driven CO-TDP-43 lines. As expected, we detected higher molecular weight species in SDS-PAGE under non-denaturing conditions in both lines tested, with increased levels in the *gl* driven CO-TDP-43 line that were absent in the non-CO-TDP-43 flies and surprisingly in *SevEP*-CO-TDP-43 lines. Interestingly, we also observed a 35 kD truncated fragment only in the codon optimized flies (Figure 3F). These bands represent the previously reported caspase cleaved C-terminal fragment that is considered to be the toxic component of TDP-43 aggregates (Liu et al., 2014; Chiang et al., 2016).

The distinctive pathology of TDP-43 mediated neurodegeneration involves its mislocalization to the cytoplasm and loss of normal nuclear expression (Neumann et al., 2006; Lee et al., 2012). Therefore, we further investigated the localization of CO-TDP-43 in neuronal cells. Figures 3G, L represents the schematic of the eye discs and the area imaged from apical and posterior views respectively. When co-stained with *Elav* and TDP-43, the eye discs showed higher nuclear and cytoplasmic expression of TDP-43 in both CO-TDP-43 lines (*gl* and *SevEP*-GAL4 driven) compared to non-CO-TDP-43 flies (Figures 3I, N vs. Figures 3J, O, K, P). In particular, the *gl* driven CO-TDP-43 flies formed punctate structures in the cytoplasm resembling cytoplasmic aggregates compared to the other transgenics (Figures 3J, O, compared to control Figures 3H, M). Overall, these observations indicate that a higher level of TDP-43 protein has the propensity to form protein aggregates *via* a gain-of-function mechanism, similar to other neurodegenerative proteins such as Tau, Aβ, and Alpha-synuclein.

### Morphological and functional disruption of photoreceptor neurons induced by codon-optimized TDP-43

**FIGURE 4:**
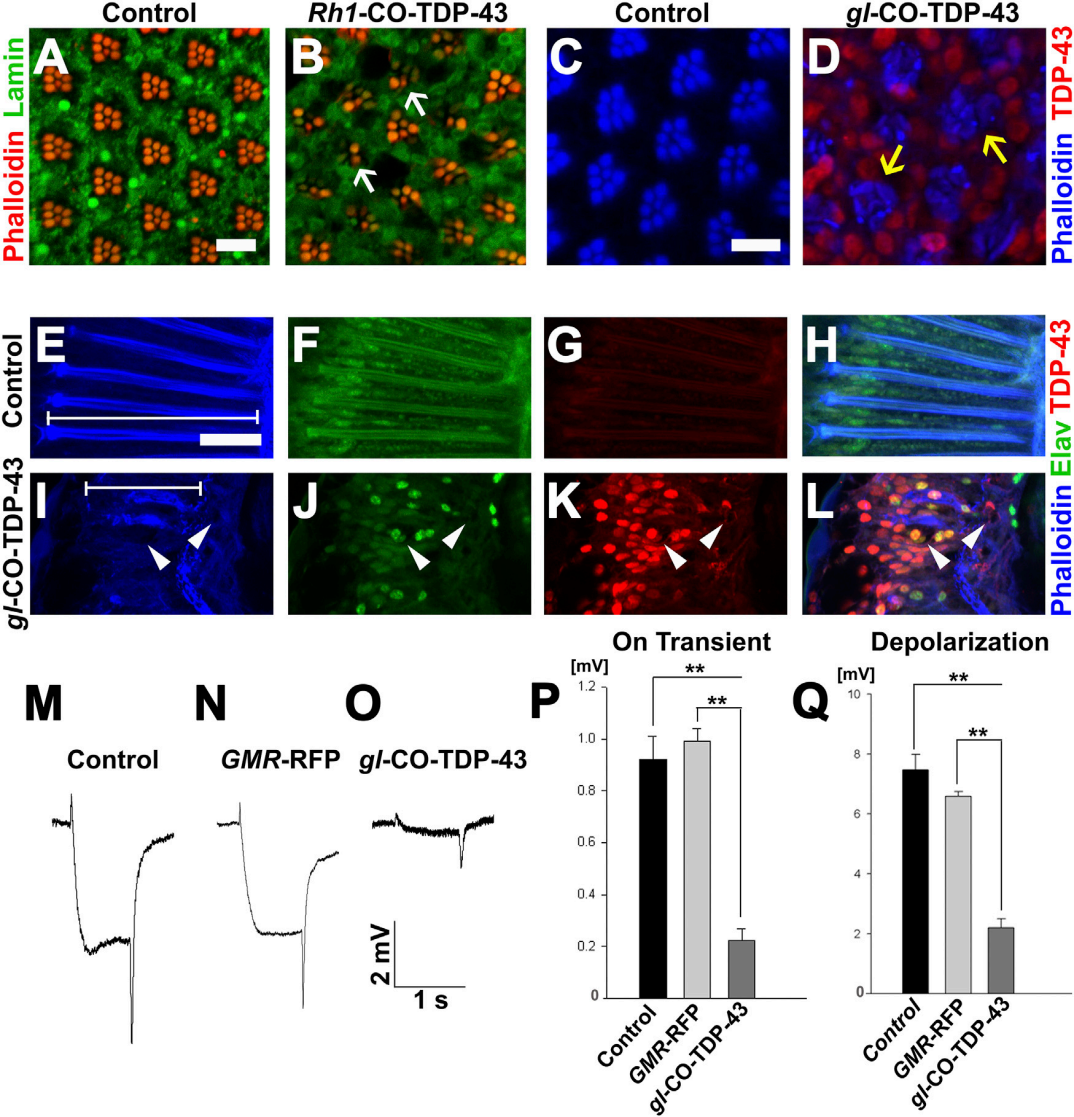
TDP-43 misexpression in adult retina causes degeneration and altered morphology of the photoreceptor neurons. **(A)** *UAS*-LacZ and **(B)** *UAS*-CO-TDP-43 expressed in the retina using *Rh1*-GAL4 that selectively expresses TDP-43 in R1-R6 photoreceptor neurons during the late pupal stage. Compared to control, the CO-TDP-43 shows loss of rhabdomeres (white arrows) and degeneration in the 7 days post-eclosion adult retina (scale bar 5 µm). The *gl*-CO-TDP-43 flies **(D)** exhibit rhabdomere separation defect and flattened structures of the rhabdomeres (yellow arrows) in 1-day post-eclosion adult compared to *GMR*-GAL4 control **(C)**, as seen in the tangential view of the retina (scale bar 5 µm). Similarly, in the longitudinal view, the *gl*-CO-TDP-43 flies **(I–L)** show altered photoreceptor morphology that appear to be shorter [white lines in (**E, I**) compared to control **(E–H)**]. The CO-TDP-43 flies also contain large vacuoles (white arrowheads) in 1-day post-eclosion adult retina (scale bar 10 µm). **(M–O)** ERG traces of wild-type control, *GMR*-RFP control and *gl*-CO-TDP-43 1-day post-eclosion adults, respectively, are shown. Quantification of the ERG response amplitude for on transient **(P)** and depolarization **(Q)**, along with the traces, show that CO-TDP-43 flies have decreased responses for both measures. For on transient effect, *n* = 15 and *p* < 0.001 between both groups **(P)**, and for depolarization effect, *n* = 15 and *p* < 0.001 between both groups **(Q)**. Genotypes: **(A)** w^1118^/+;*Rh1*-GAL4/+; UAS-LacZ/+, **(B)** w^1118^/+;*Rh1*-GAL4/UAS-TDP-43^CO^;+, **(C)** Canton S, **(D)** w^1118^;*gl*-TDP-43^CO^/+; +, **(E–H)** Canton S, **(I–L)** w^1118^;*gl*-TDP-43^CO^/+; +, **(M)** Canton S, **(N)** w^1118^;*GMR*-GAL4/+; UAS-RFP/+, **(O)** w^1118^;*gl*-TDP-43^CO^/+; +.

Based on severe retinal phenotypes observed with toxic aggregates of TDP-43 protein, we further investigated the internal cellular morphology of the photoreceptor neurons. We utilized another eye-specific driver, *Rh1*-GAL4, which is expressed in R1–R6 neurons starting in late pupal stage and persisting throughout adulthood (Chyb et al., 1999). Unlike the *GMR*-GAL4, *SevEP*-GAL4 or *gl* direct fusion lines, this driver allowed us to examine adult-onset expression of TDP-43. In 7-day post-eclosion CO-TDP-43 flies (Figure 4B), we observed a degenerative phenotype in the adult retina marked by the loss of rhabdomere structures and vacuolization compared to the control flies (Figure 4A). Comparatively, using the *gl* direct fusion CO-TDP-43 line, we observed the degenerative phenotype as early as 1-day in post-eclosion flies. The *gl*-CO-TDP-43 flies (Figure 4D) exhibited an altered morphology of the photoreceptor neurons, which appeared to be flattened and had a disruption in rhabdomere separation compared to control flies (Figure 4C) when visualized in the tangential view of the adult retina. Examination of the longitudinal view of the adult retina showed a marked reduction in thickness and shorter photoreceptor length compared to controls (marked by white lines in Figures 4E, I). In addition, we found that these photoreceptor neurons were accompanied by large vacuolar structures, and co-staining with *Elav* revealed that TDP-43 was localized both in the nucleus and in the cytoplasm (Figures 4I–L) compared to control flies (Figures 4E–H).

To investigate the physiological functions of these photoreceptor neurons, we used electroretinogram (ERG) recordings to measure the functionality of active photoreceptor neurons by measuring their response to light stimulus (Dolph et al., 2011). In fact, *Drosophila* neurodegeneration models overexpressing Tau and Alpha-synuclein exhibited degenerative pathology in the fly retina along with neuronal dysfunction, as detected by ERG recording (Chouhan et al., 2016). Using this technique, we investigated whether CO-TDP-43 misexpression affects neuronal functionality compared to wild-type and *GMR*-RFP controls (Figures 4M–O). The *gl*-CO-TDP-43 flies demonstrated a reduction in both the amplitude of ERG in “on transient” and evoked depolarization at 1-day post-eclosion (Figures 4P, Q). These effects were not observed with either control. Taken together, these results strongly suggest that CO-TDP-43 misexpression causes structural and functional degenerative phenotypes in the adult retina.

### Codon-optimized wild-type TDP-43 misexpression disrupts cellular lysosomal and autophagic processes

**FIGURE 5:**
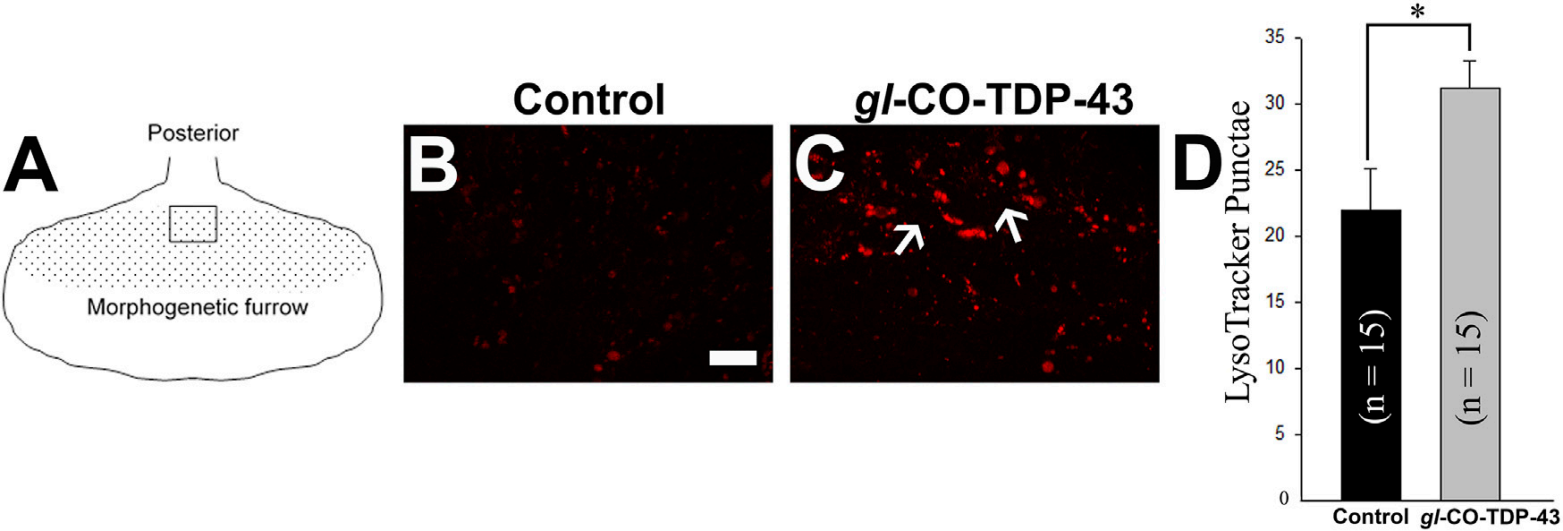
TDP-43 misexpression leads to increased lysosomal vacuoles positive for autophagy markers. **(A)** Schematic of the third instar larvae and the area imaged. Live staining of the LysoTracker dye shows increases in lysosomal punctae in *gl*-CO-TDP-43 flies **(C)** compared to control **(B)**. Scale bar equals 10 µm. **(D)** shows quantification of **(B, C)**, *n* = 15 and *p* = 0.02. Genotypes: **(B)** Canton S, **(C)** w^1118^/+;*gl*-TDP-43^CO^/+; +.

An autophagic dysfunction has been implicated in many neurodegenerative diseases, including ALS (Wong and Cuervo, 2010; Brady et al., 2011; Sasaki, 2011). Therefore, the presence of large vacuolar structures in the adult retina of CO-TDP-43 flies (see Figure 4) led us to investigate if these vacuoles could possibly be a representation of autophagic intermediates. We performed live imaging of larval eye discs using LysoTracker to detect lysosomes and other acidic organelles, such as autophagosomes, that typically increase in number and/or size during the later stages of autophagy. Figure 5A represents the schematic of the eye discs and the area imaged. Upon CO-TDP-43 misexpression, we detected a significantly larger number of acidic punctae compared to control flies (Figure 5C, white arrows, compared to control in Figure 5B; Figure 5D), suggesting increases in autophagosomes due to elevated levels of TDP-43.

To further characterize the large vacuoles, we coexpressed CO-TDP-43 and a tagged autophagy protein, Atg5-GFP, which is responsible for the formation of the autophagosomes. We found that these vacuoles were positive for both Atg5 and TDP-43 (Supplementary Figures S5A–D). During autophagy, autophagosomes merge with lysosomes to become autolysosomes and are acidified to degrade proteinaceous waste materials (Zhang et al., 2013). To determine if the autophagosomes observed were mature and functional autolysosomes, we used the Atg8-mCherry-GFP tandem reporter to assay the relative acidity of the autophagosome/autolysosomes. Atg8-mCherry-GFP is a tandem reporter that detects Atg8, which is localized in autophagic intermediates, and a pH-sensitive GFP that only emits a signal at a neutral pH (Filimonenko et al., 2007). This is a useful tool to help understand whether the large vacuoles observed in our CO-TDP-43 flies were autophagic as well as acidic, which is characteristic of autophagic intermediates. We coexpressed CO-TDP-43 with the tandem reporter and found that the larger punctae were positive for Atg8-mCherry, while only a subset of the relatively smaller punctae were stained with GFP, indicating non-acidic compartments (Supplementary Figures S5E–G). In contrast, the majority of the punctae that were larger in size were only fluorescent for Atg8-mCherry (Supplementary Figures S5E–G), indicating more acidic and mature autolysosomes. These results suggest that misexpression of CO-TDP-43 leads to increased acidic lysosomal vacuoles that are indicative of autophagic dysfunction.

## Discussion

To date, little is known about the exact mechanism of action of TDP-43 mediated toxicity. Here, we report a novel transgenic *D. melanogaster* resource to better understand TDP-43 mediated neurodegeneration. There is great potential for the codon optimized TDP-43 model, as it exhibits robust and sensitive phenotypes ideal for genetic manipulations that allow us to understand its pathogenic mechanisms in an *in vivo* system. Our results suggest that this model has important utility in understanding the TDP-43 mediated pathology in neurodegenerative disorders.

Previous studies using wild-type TDP-43 fly models helped us understand how the misfolded proteins contributed to neurotoxicity and eventual neurodegeneration (Li et al., 2010). However, there are several limitations associated with them. The reported models did not show strong phenotypes, and conclusions drawn from the fly studies depend heavily upon lines containing pathogenic variants (Lu et al., 2009; Li et al., 2010; Ritson et al., 2010; Voigt et al., 2010; Miguel et al., 2011; Langellotti et al., 2016; Chang and Morton, 2017). Similar to published reports, we were previously unable to show any robust phenotypes with the wild-type human transgenic TDP-43 flies (Choksi et al., 2014). As a majority of ALS and FTLD cases do not carry known pathogenic mutations, it is critical to understand the mechanism by which the wild-type TDP-43 drives the disease. Compared to previously reported wild-type TDP-43 models, CO-TDP-43 flies exhibit robust eye, wing, and bristle phenotypes, mirroring disease-specific characteristics of TDP-43 (summarized in Table 1). Our findings are in line with previous studies that associated pathogenic mutations in TDP-43 to severely damaged sensory neurons, affecting both the central and peripheral nervous systems in patients (Camdessanche et al., 2011). The robust phenotypes observed in our study are indicative of cellular dysfunction and death and are probable markers for neurodegenerative models. Using genetic and molecular approaches to analyze the mechanisms underlying TDP-43 mediated phenotypes in the eye or the wing may elucidate plausible therapeutic targets of TDP-43.

**TABLE 1:** Gain-of-function Drosophila models for TDP-43. A comparison of human wild-type TDP-43 transgenic gain-of-function models and codon-optimized TDP-43 models for disease-specific findings.

Gain-of-function *Drosophila* models for TDP-43											
	*CO-TDP-43 (this paper)*	Lu et al. (2009)	Voigt et al. (2010)	Hanson et al. (2010)	Li et al. (2010)	Ritson et al. (2010)	Estes et al. (2011)	Guo et al. (2011)	Miguel et al. (2011)	Chang and Morton (2017)	Pons et al. (2017)
Transgenic model	human CO- TDP-43	human wt TDP-43	Synthetic wt TDP-43	human wt TDP-43	human wt TDP-43	human wt TDP-43	human wt TDP-43	human wt TDP-43	human wt TDP-43	human wt TDP-43	human wt TDP- 43 with TBPBR expression
Eye phenotype	**+**	**-**	**-**	**+** (age dependent phenotype)	**+** (age dependent phenotype)	**-**	**+** (at increased temp 29 °C)	**-**	**+**	**-**	**+**
Sensory tissue phenotype	**+**	**+**	**-**	**-**	**-**	**-**	**-**	**-**		**-**	**-**
Cytoplasmic mislocalization	**+**	**-**	**-**	**-**	**+**	**-**	**+**	**-**	**+**	**-**	**-**
High-molecular weight/oligomeric species	**+**	**-**	**-**	**-**	**-**	**-**	**-**	**-**	**-**	**-**	**-**
Cellular aggregation	**+**	**-**	**-**	**-**	**+**	**-**	**+**	**-**	**-**	**-**	**-**
Autophagic upregulation	**+**	**-**	**-**	**-**	**-**	**-**	**-**	**-**	**-**	**-**	**-**

In addition, our CO-TDP-43 transgenic model affected multiple different cell types in the fly retina, as evident by depigmentation of pigment cells, irregularities in interommatidial bristle cells, disruption in rhabdomeres morphology, disruption of photoreceptor neuron morphology, and necrosis of the cone cells. The effects of neurodegenerative proteins on the *Drosophila* eye can be diverse. For example, in a polyglutamine-expanded human huntingtin transgenic model, the expanded huntingtin protein was shown to form nuclear inclusions and cause severe degeneration of photoreceptor cells (Jackson et al., 1998). Furthermore, the human wild-type Tau transgenic model showed abnormal polarity and some rhabdomere loss, mostly affecting the cone cells and ommatidial architecture (Jackson et al., 2002; Ambegaokar and Jackson, 2011). It is worth noting that we observed a more severe eye-phenotype in our *gl*-CO-TDP-43 line compared to *SevEP*-GAL4 driven CO-TDP-43. This is perhaps not surprising considering that *SevEP*-GAL4 is expressed only in a subset of photoreceptor neurons. Based on our observations using a wide array of eye-specific drivers, we find that TDP-43 pathology is not restricted to photoreceptor neurons but is most likely widespread among different cell types in the *Drosophila* retina. In fact, TDP-43 is known to be present and shows disease-related pathology across different types of cells both in humans and in animal models (Mackenzie and Rademakers, 2008; Wegorzewska et al., 2009). Moreover, a *Drosophila* model of TDP-43 has been shown to exhibit individual responses in motor neurons and glial cells (Estes et al., 2011). The CO-TDP-43 flies would therefore serve as an ideal genetic resource to pursue in-depth investigations that determine the morphological effects of TDP-43 in diverse cell types.

There is growing evidence of increased levels of TDP-43 protein in the plasma of patients with ALS (Foulds et al., 2008). More recently, Ren and colleagues showed that both total TDP-43 and phosphorylated TDP-43 levels have increased in plasma and cerebrospinal fluid of patients with ALS (Ren et al., 2021). This translocation of TDP-43 aggregates from the nucleus to the cytoplasm to form insoluble inclusions remains a salient feature of this disease. In these inclusions, the aggregated forms of TDP-43 protein are hyperphosphorylated at Ser409/410 (Choksi et al., 2014). Our CO-TDP-43 transgenics yield a higher level of TDP-43 and display more robust and toxic phenotypes. This is unsurprising, considering that there have been reports in both sporadic and familial cases of FTLD of increased TDP-43 expression in patient brain tissues (Mishra et al., 2007; Gitcho et al., 2009). In addition, in our transgenic fly models, we find aggregated and hyperphosphorylated TDP-43 (Ser409/410) mislocalized in the cytoplasm which represents the major pathological hallmarks in the ALS/FTLD cases (Neumann et al., 2009).

Protein misfolding is one of the salient features of neurodegenerative diseases. Hyperphosphorylated and aggregated forms of Tau protein are found in the brains of patients with Alzheimer’s Disease (Wu et al., 2022). Similarly misfolded and aggregated Alpha-Synuclein deposits are found in individuals with Parkison’s Disease and in Dementia with Lewy Bodies (Sanderson et al., 2020). The reason for the progressive accumulation of these misfolded proteins in the cytoplasm is unclear. This is particularly true for TDP-43 protein which in the disease-free state is localized in the nucleus as a DNA/RNA binding protein (Ayala et al., 2008; Winton et al., 2008; Lee et al., 2012). One reason for its mislocalization to the cytoplasm could be the high-expression of TDP-43. Increased levels of TDP-43 lead to self-aggregation and accumulation within the stress-granules (Gao et al., 2018). This is also accompanied by post-translational modifications such as hyperphosphorylation at Ser409/410 residues that further promote the formation of detergent insoluble aggregates. Indeed, in a recent study (Chou et al., 2018) it has been found that TDP-43 pathology causes the disruption of the nucleocytoplasmic transport machinery causing the aggregation and mislocalization of the nucleoporins and transport factors. Other studies have shown that TDP-43 can impair autophagic clearance leading to the formation of inclusion bodies in ALS and FTLD patients that are positive for autophagy markers and accumulate TDP-43 oligomers (de Boer et al., 2021). In our CO-TDP-43 models we not only observe a 2-fold increase in the levels of total TDP-43 but also a substantial enhancement of phospho-TDP43 (Ser409/410). Both are consistent features of sporadic and familial forms of ALS. Although further studies are required, it is possible that in this CO-TDP-43 transgenics there could be a disruption of nucleocytoplasmic protein transfer pathways and/or autophagic impairment leading to a progressive accumulation of cytoplasmic aggregates of TDP-43.

CO-TDP-43 induced neurotoxicity was validated by the electrophysiological readouts in our study, which showed that toxic aggregates of wild-type TDP-43 reduced functional activity in photoreceptor neurons in the adult eye. The cytoplasmic mislocalization and presence of toxic TDP-43 aggregates have been well characterized in human patient samples of ALS/FTLD (Geser et al., 2009; Ritson et al., 2010; Miguel et al., 2011; Lee et al., 2012; Chang and Morton, 2017). While *in vitro* studies have shown disease-specific mutant or truncated TDP-43 can form toxic aggregates of oligomeric species, very few studies in wild-type TDP-43 animal models have demonstrated a similar robust production of TDP-43 aggregates (Johnson et al., 2009; Couthouis et al., 2011; Guo et al., 2011; Lee et al., 2012; Choksi et al., 2014). Our study is unique in that it showed a similar accumulation of toxic aggregates with wild-type human TDP-43 protein. Hence, there is a possibility that TDP-43 has a dosage-dependent effect on its propensity to form toxic aggregates. Several groups have further shown that cytoplasmic mislocalization of TDP-43 causes neuronal toxicity (Shan et al., 2009; Barmada et al., 2010). Previously, we have been able to show such robust mislocalization of TDP-43 only with disease-specific mutant hTDP-43 Q331K flies (Choksi et al., 2014). In keeping with these observations, the disease-specific, dysfunctional phenotypes that we observed with misexpression of wild-type TDP-43 in our codon optimized model offer a great resource to study the cellular processes that could be involved with ALS/FTLD.

Lastly, in our CO-TDP-43 model, we observed an increase in acidic vacuoles, as evident by lysotracker staining, that are positive for autophagic proteins ATG5 and ATG8 known to be involved in the formation of early and late stage autophagosomes. At this stage our data is too preliminary to infer whether these vacuoles imply an autophagic induction to clear misfolded TDP-43 aggregates or a complete blockage of this process. Autophagic impairment is a common feature of many neurodegenerative diseases, including Alzheimer’s disease, Parkinson’s disease, Huntngton’s disease, and ALS (Wong and Cuervo, 2010; Sasaki, 2011). In patients with sporadic ALS accumulation of autophagosomes was observed in the spinal cord tissues (Sasaki, 2011). Recent studies have reported that an inhibition of the ubiquitin proteasome system and autophagy led to increased TDP-43 aggregation and toxicity (Brady et al., 2011). In addition, p62, which is a part of the ubiquitin proteasome system, has been identified to directly bind with TDP-43, and its overexpression can reduce TDP-43 aggregation (Tanji et al., 2012).

All of this evidence strongly suggests that misfolded TDP-43 aggregates might play a role in impairing autophagic processes thereby causing more toxic aggregates to accumulate. Interestingly, our previous studies in *Drosophila* Tauopathy models have shown that misexpression of Tau leads to severe autophagic dysfunction by decreasing lysosomal acidification (Bakhoum et al., 2014). Further experiments need to be done in our CO-TDP-43 transgenics to investigate the status of autophagy in response to the formation of toxic TDP-43 protein aggregates making this an excellent resource for investigating the underlying mechanistic pathways.

However, our model has a few limitations. A significant aspect to consider for future studies is whether the intrinsic structure and stability of the TDP-43 protein changes due to codon-optimization. There are numerous reports that phosphorylation of TDP-43 results in its mislocalization in the cytoplasm, thus hindering its ability to shuttle back to the nucleus and bind to nucleic acids (Chou et al., 2018; Nonaka and Hasegawa, 2018). Interestingly, the structural changes that accompany enhanced TDP-43 expression or post-translational modifications (phosphorylation, oxidation, acetylation, sumoylation) still remain largely unexplored making this a significant area of future research (François-Moutal et al., 2019). Also, the impact of the endogenous *Drosophila* TBPH on the codon-optimized human TDP-43 needs to be examined. Several studies have shown that the knock-down of TBPH in fruit flies causes locomotor deficits. The compensation of misexpressed codon-optimized human TDP-43 by endogenous TBPH remains an interesting area of study for a comprehensive understanding of the pathways leading to neurodegeneration. The locomotor and longevity assays following the pan-neuronal expression of CO-TDP-43 is another important area that needs substantial research to open new vistas for developing future therapeutics. Despite these limitations, our codon-optimized model displaying a robust neurodegenerative phenotype compared to the non-codon-optimized transgenics is a promising start. In this study, we have shown that unlike other fly models that rely on pathogenic mutations, CO-TDP-43 model recapitulates many of the known phenotypes of TDP-43 proteinopathies observed in humans.

Furthermore, TDP-43 is an RNA-binding protein that is involved with RNA metabolism and regulation. As a result, much effort has been devoted to identifying the RNA targets of TDP-43 using cell culture models, animal models, and ALS and FTLD patient brain samples. Recently, TDP-43 was shown to bind approximately 30% of the mouse transcriptome, identifying a vast number of possible interactors that can associate with TDP-43 to regulate RNA processing and splicing (Polymenidou et al., 2011; Tollervey et al., 2011). Many of these putative modifiers bind the UG-rich sequence at introns of TDP-43 (Bhardwaj et al., 2013). In this context, our human CO-TDP-43 expressing fly model provides an *in vivo* platform to characterize and validate some of these modifiers to better understand the TDP-43-dependent disease mechanism in ALS/FTLD. In conclusion, the robust phenotypes observed in the external organs of the eye, wing, and notum of these flies can be scored easily, offering an excellent model for high-throughput screens of modifiers genes that will help elucidate the molecular mechanism of toxicity due to TDP-43. Targeted genetic screens that identify effectors of TDP-43 will allow us to further identify and pursue novel mechanisms for disease pathology.

## Data Availability

The original contributions presented in the study are included in the article/Supplementary Material, further inquiries can be directed to the corresponding authors.
