# Supplementary material for: Codon-optimized TDP-43 mediates neurodegeneration in a Drosophila model of ALS/FTLD
Source: Front Genet. 2023 Mar 9;14:881638. doi: 10.3389/fgene.2023.881638 (PMC10034021; doi:10.3389/fgene.2023.881638)
Supplement: Supplementary file 2 [file Image5.pdf]

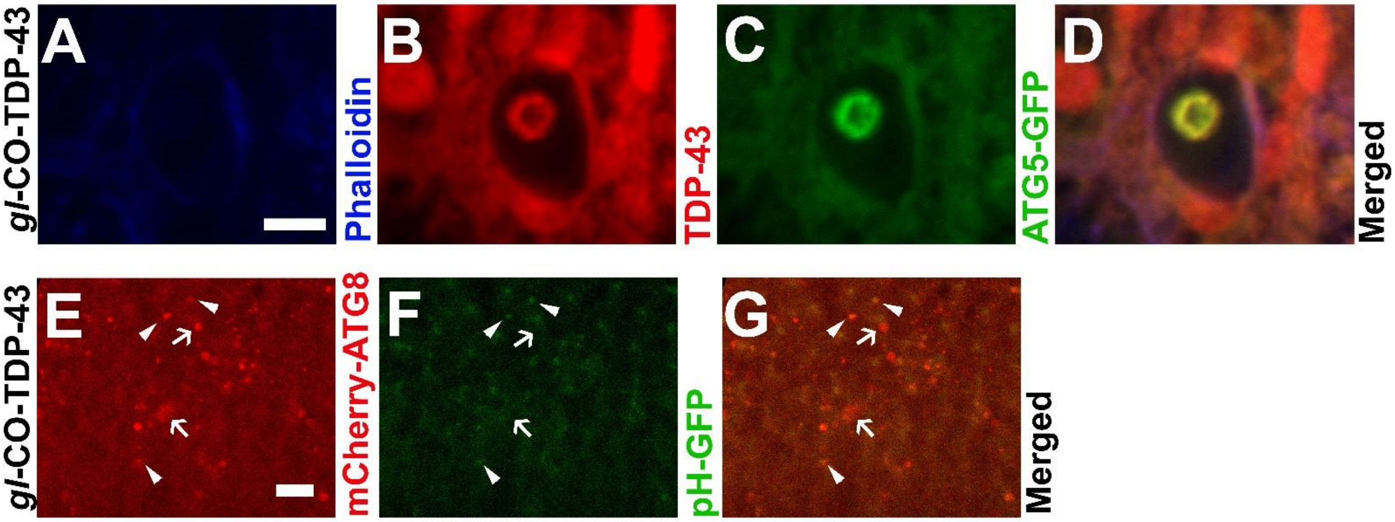

Figure S5. Misexpression of codon optimized TDP-43 exhibits large vacuoles positive for autophagy markers. (A-D) Coexpression of *gl*-CO-TDP-43 and the autophagic protein Atg5-GFP shows that the large vacuoles present in the 1-day post-eclosion adult retina are positive for Atg5 (scale bar 5 µm). **(E-G)** Another autophagic marker was coexpressed with *gl*-CO-TDP-43, Atg8-mCherry-GFP, which is pH sensitive and only expresses GFP at a higher pH content. The CO-TDP-43 expressed flies show that few of the relatively smaller punctae were positive for both Atg8-mCherry and GFP (arrowheads), while a majority of the larger punctae were only fluorescent for Atg8-mCherry (white arrows), indicating more acidic punctae (scale bar 10 µm). Genotypes: **(A-D)** w^{1118}, *GMR*-GAL4/ w^{1118};*gl*-TDP-43^{CO}/+;UAS-Atg5-GFP/+, **(E-G)** w^{1118}, *GMR*-GAL4/ w^{1118};*gl*-TDP-43^{CO}/UAS-Atg8-mCherry-GFP;+.
