# Supplementary material for: Codon-optimized TDP-43 mediates neurodegeneration in a Drosophila model of ALS/FTLD
Source: Front Genet. 2023 Mar 9;14:881638. doi: 10.3389/fgene.2023.881638 (PMC10034021; doi:10.3389/fgene.2023.881638)
Supplement: Supplementary file 3 [file Image4.pdf]

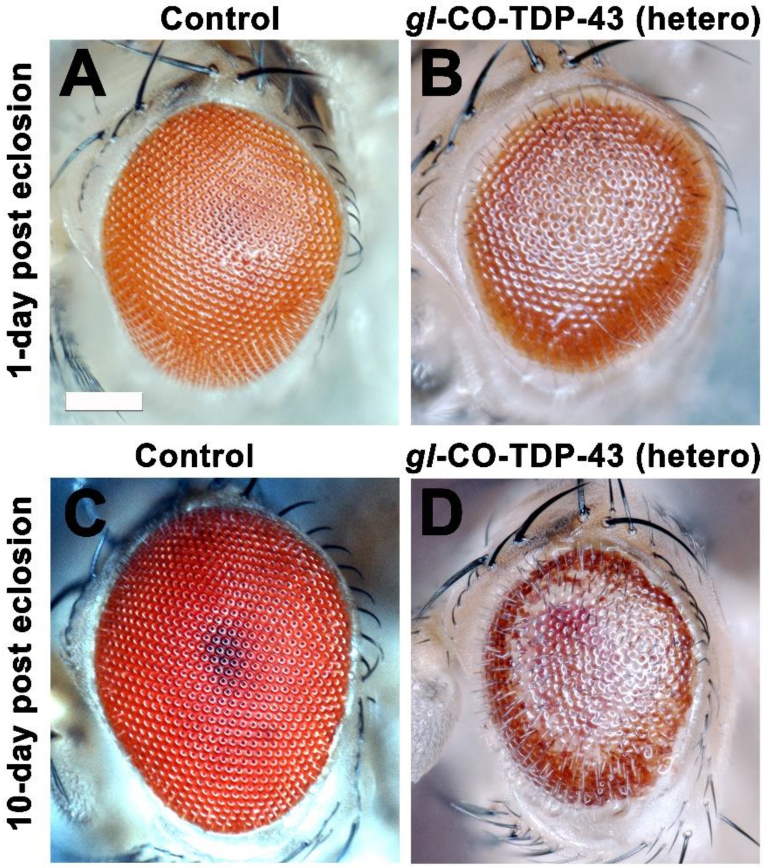

Figure S4. Heterozygous misexpression of codon optimized TDP-43 induces age dependent phenotype in the external eye. Transgenic flies expressing heterozygous CO-TDP-43 show mild eye phenotypes at 1-day post-eclosion **(B)** and severe eye phenotype at 10-day post-eclosion **(D)** exhibiting an age dependent affect compared to age matched controls **(A and C)**. Scale bar: 100 nm. Genotype: **(A)** Canton S, **(B)** w^{1118}/+;*gl*-TDP-43^{CO}/+;+, **(C)** Canton S **(D)** w^{1118}/+;*gl*-TDP-43^{CO}/+;+.
