# Supplementary material for: Codon-optimized TDP-43 mediates neurodegeneration in a Drosophila model of ALS/FTLD
Source: Front Genet. 2023 Mar 9;14:881638. doi: 10.3389/fgene.2023.881638 (PMC10034021; doi:10.3389/fgene.2023.881638)
Supplement: Supplementary file 4 [file Image2.pdf]

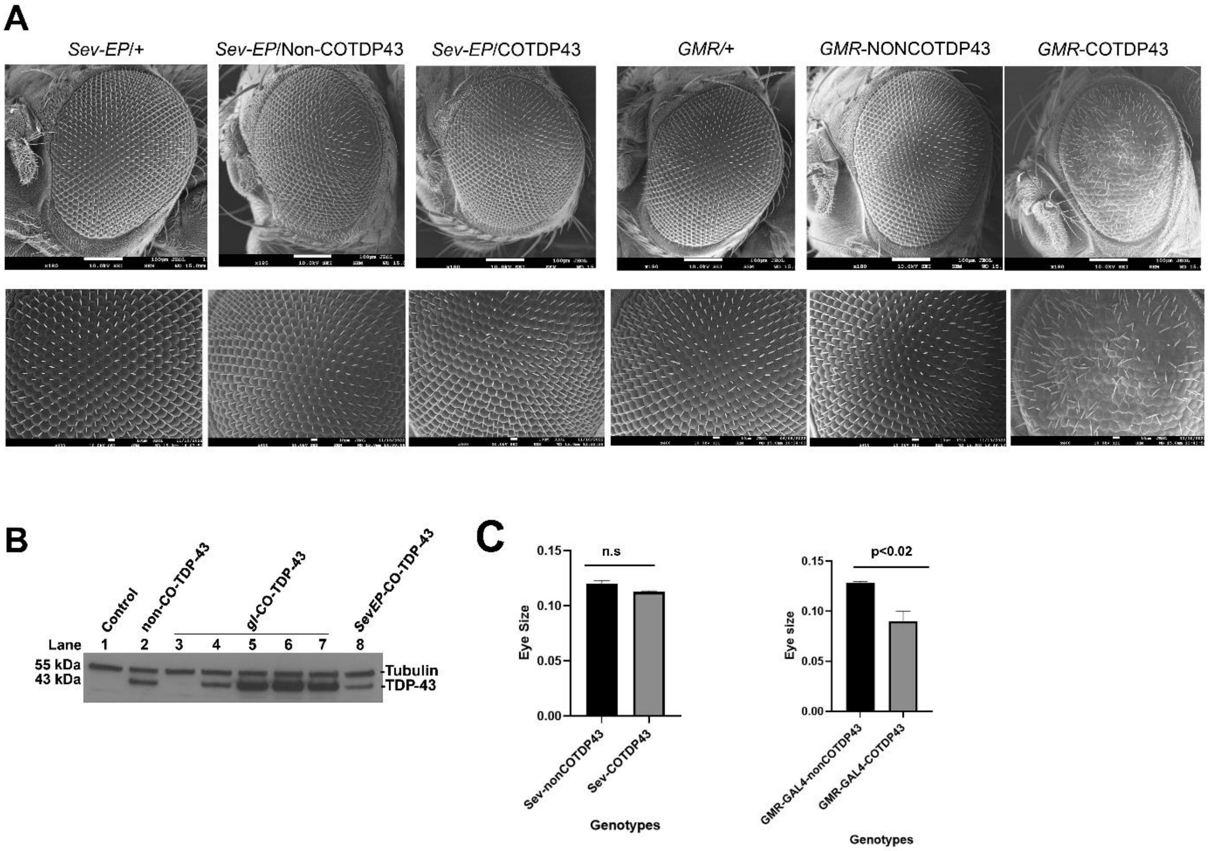

Figure S2. Misexpression of codon optimized TDP-43 using eye-specific drivers show robust eye phenotypes and increased phosphorylation of TDP-43 protein. (A) SEM images display a significant reduction in size and a “rough-eye” phenotype in the *GMR*-GAL4 driven CO-TDP-43 transgenics compared to non-CO-TDP-43 transgenic lines. This effect is not that pronounced in the *SevEP*-GAl4 driven transgenic lines. **(B)** Western blot analysis comparing the total TDP-43 levels in eyes from human wild-type TDP-43 flies (lane 2) to the different generated codon optimized TDP-43 lines (lane 3-8) and control flies (lane 1). Among the different codon optimized TDP-43 lines, gl-TDP-43CO3, gl-TDP-43CO4 and gl-TDP-43CO5 (lane 5, 6 and 7 respectively) have the highest expression of TDP-43, almost a 2-fold increase compared to human wild-type TDP-43. Codon optimized TDP-43 expression driven with a selective R7 and R8 photoreceptor neuron driver, SevEP-GAL4, did not show increased expression of the total protein (lane 8). β-tubulin is presented as a loading control. **(C)** Bar graphs representing a difference in eye-sizes between CO-TDP-43 and non-CO-TDP-43 transgenics. This is significant in *GMR*-GAL4 driven CO-TDP-43 vs non-CO-TDP-43 flies. *p<0.02 by unapired t-test.
