# Supplementary material for: Codon-optimized TDP-43 mediates neurodegeneration in a Drosophila model of ALS/FTLD
Source: Front Genet. 2023 Mar 9;14:881638. doi: 10.3389/fgene.2023.881638 (PMC10034021; doi:10.3389/fgene.2023.881638)
Supplement: Supplementary file 5 [file Image3.pdf]

Not1 site-*Drosophila Kozak sequence*-**ATG**-codon optimized TDP-43-**Stop codon**-***Xba1 site***

TCC***GCGGCCGC*****ATCAAC****ATG**AGCGAATACATTCGGGTCACGGAGGATGAGAACGATGAGCCCATC

GAGATCCCTTCGGAGGACGATGGCACGGTTCTGCTCTCCACCGTTACCGCCCAGTTTCCGGGAGCG

TGCGGTCTGAGGTATCGTAATCCCGTGTCCCAGTGCATGAGGGGCGTGCGCCTGGTCGAGGGCAT

ACTGCACGCTCCTGACGCTGGCTGGGGCAATTTGGTGTACGTGGTGAACTACCCGAAGGATAACAA

GAGGAAGATGGATGAGACAGACGCCTCCAGTGCGGTGAAGGTGAAACGAGCGGTCCAAAAGACTA

GTGACCTTATAGTTCTGGGACTTCCGTGGAAAACGACAGAGCAGGATTTGAAAGAATACTTCTCGAC

CTTCGGAGAGGTATTGATGGTGCAAGTGAAGAAGGATCTTAAGACTGGCCATAGTAAAGGCTTTGGT

TTCGTACGCTTCACGGAGTATGAAACCCAAGTGAAGGTTATGTCGCAGCGCCATATGATTGACGGAC

GTTGGTGCGATTGTAAGCTGCCCAACAGCAAGCAGAGTCAAGATGAGCCCCTCCGCAGCCGAAAAG

TGTTCGTCGGTCGCTGTACTGAGGACATGACAGAGGACGAACTCCGAGAATTCTTTTCCCAATATGG

TGACGTTATGGACGTGTTCATCCCGAAACCTTTTCGGGCCTTCGCATTCGTCACCTTTGCTGACGAC

CAGATAGCACAGTCCCTCTGCGGAGAAGATCTGATCATTAAGGGCATCAGCGTGCACATCTCCAACG

CTGAACCAAAGCACAATAGCAACCGTCAGTTGGAGCGCTCGGGACGGTTCGGCGGTAATCCCGGTG

GCTTCGGTAACCAGGGCGGATTCGGCAATAGCCGGGGTGGAGGTGCGGGACTGGGAAACAACCAG

GGTTCGAATATGGGCGGTGGCATGAACTTTGGAGCCTTTAGCATCAACCCAGCTATGATGGCGGCT

GCACAGGCCGCGTTGCAATCGAGTTGGGGTATGATGGGCATGCTGGCATCCCAGCAAAATCAGAGT

GGACCCTCCGGCAATAACCAGAATCAAGGCAATATGCAGCGCGAACCAAATCAGGCCTTTGGCTCC

GGAAACAACTCGTACTCCGGCAGCAATTCGGGCGCAGCCATTGGATGGGGCAGCGCCTCCAATGC

CGGTAGCGGCAGCGGCTTCAACGGCGGCTTTGGCAGTTCGATGGATAGTAAGAGCTCGGGTTGGG

GCATG**TAA*****TCTAGA*****TAA**

Figure S3: Sequence of codon optimized human wild-type TDP-43.
