# Supplementary material for: Codon-optimized TDP-43 mediates neurodegeneration in a Drosophila model of ALS/FTLD
Source: Front Genet. 2023 Mar 9;14:881638. doi: 10.3389/fgene.2023.881638 (PMC10034021; doi:10.3389/fgene.2023.881638)
Supplement: Supplementary file 7 [file Image1.pdf]

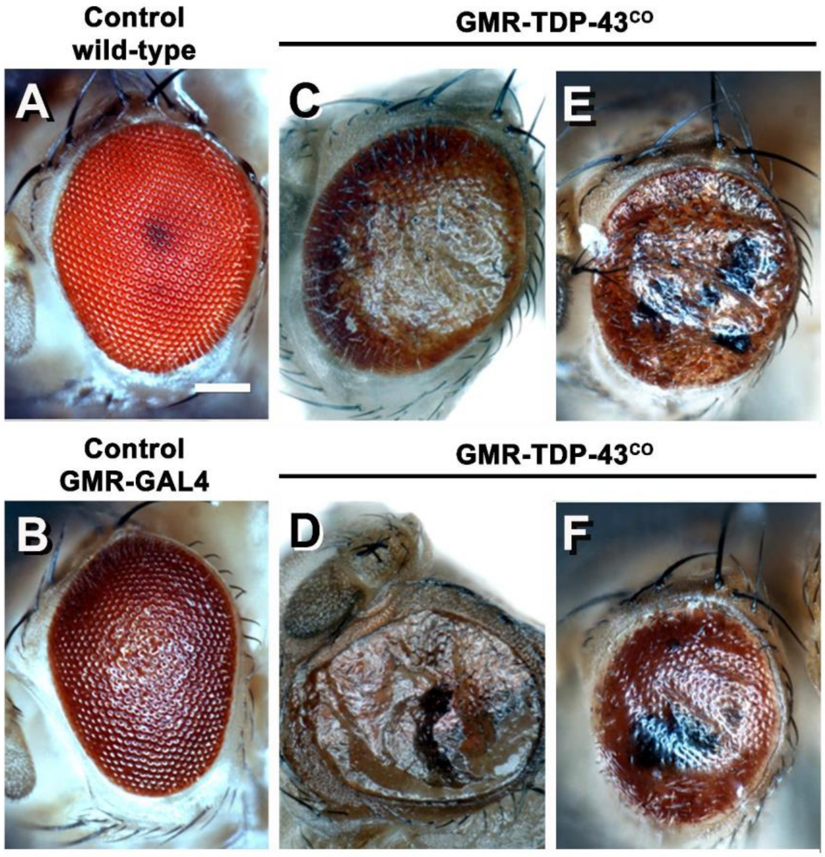

Figure S1. Misexpression of codon optimized TDP-43 using GMR-GAL4 promoter induces robust phenotype in the external eye. (C-F) Transgenic flies expressing CO-TDP-43 showing severe eye phenotypes compared to controls (A and B). Scale bar: 100 nm. Genotype: **(A)** Canton S, **(B)** w1118/+;GMR-GAL4/+;+; **(C and E)** w1118/+; GMR-GAL4/UAS-TDP-43^{CO};+, **(D and F)** w1118/+; GMR-GAL4/+;UAS-TDP-43^{CO}/+.
